# Juxta Cortical Tibia Metastatic Deposition in Gastric Cancer: A Case Report

**DOI:** 10.1155/2012/695627

**Published:** 2012-03-21

**Authors:** Majid Sajjadi Saravi, M. Hossein Karimi Nasab, Masoud Shayeste Azar, Ghasem Janbabai, Mehrdad Taghipour, S. Omid Emadian Saravi, Fariborz Eshghi

**Affiliations:** ^1^Department of Orthopedic Surgery, School of Medicine, Mazandaran University of Medical Sciences, 4813894393 Sari, Iran; ^2^Department of Oncology and Hematology, Cancer Research Center, School of Medicine, Mazandaran University of Medical Sciences, 4813894393 Sari, Iran; ^3^Student Research Committee, Cancer Research Center, School of Medicine, Mazandaran University of Medical Sciences, 4813894393 Sari, Iran; ^4^Department of Pathology, School of Medicine, Mazandaran University of Medical Sciences, 4813894393 Sari, Iran; ^5^Department of Surgery, School of Medicine, Mazandaran University of Medical Sciences, 4813894393 Sari, Iran

## Abstract

We report a 41 years old man with rapidly growing and tender lump on the anteromedial surface of tibia. The patient had the history of gastrectomy and gastrojejunostomy due to gastric carcinoma. On admission, the Simple X-ray of lower extremity disclosed a slight thinning of the anterior cortex of tibia without cortical destruction. The whole-body bone scan with ^99m^TC MDP revealed activity of lesion in all 3 phases. The histopathological evaluation showed an infiltration of bone with tumor cells. Review No the literature revealed in previous cases of skeletal metastasis from gastric cancer in the tibia like this.

## 1. Introduction

Gastric cancer has not been recognized as a number of the group of common bone invaders consisting of lung, prostate, breast, thyroid and kidney cancers [1]. Moreover, metastatic bony lesions tend to occur in axial skeleton or the roots of extremities and their occurrence below elbow or knee is quite rare [2].

## 2. Case Report

**Figure 1:**
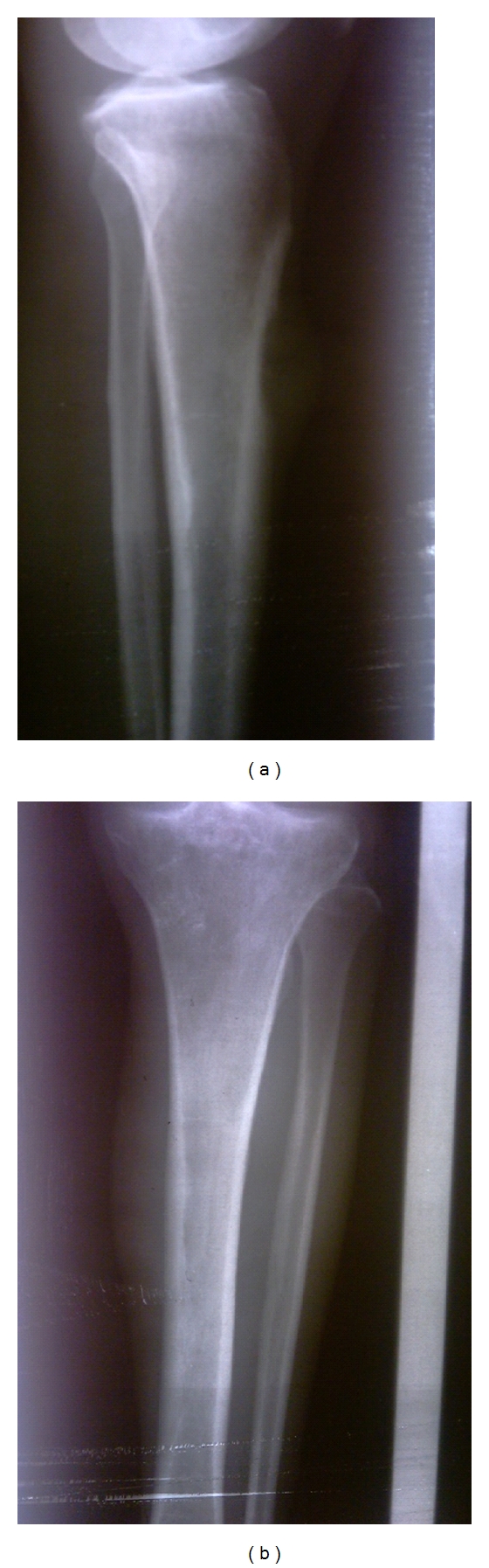
Simple X-ray of lower extremity. Slight thinning of the anterior cortex without cortical destruction.

**Figure 2:**
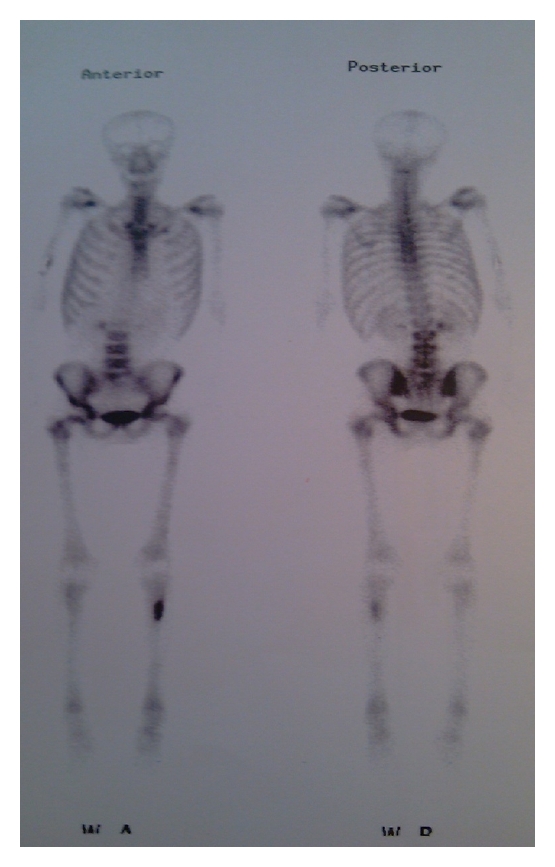
Whole-body bone scan with Tc-99 m MDP, the lesion showed activity in all 3 phases and was the only site of skeletal involvement.

**Figure 3:**
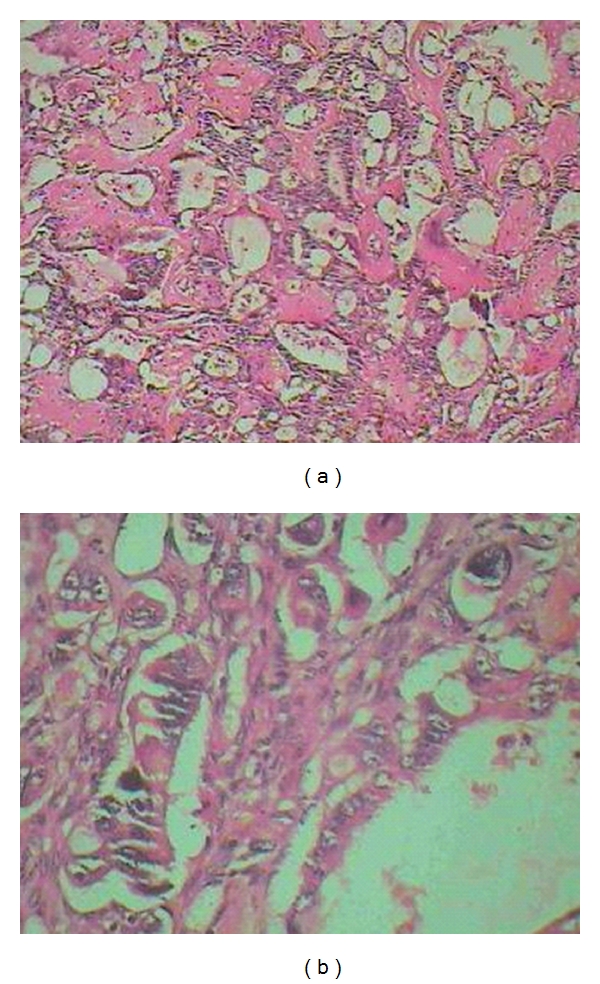
Histopathological section of metastatic tumor. (a) Low power field: the tumor infiltrated bone as atypical glands. (b) High power field: atypical gastric glands lined by pleomorphic hyperchromatic tumor cells.

**Figure 4:**
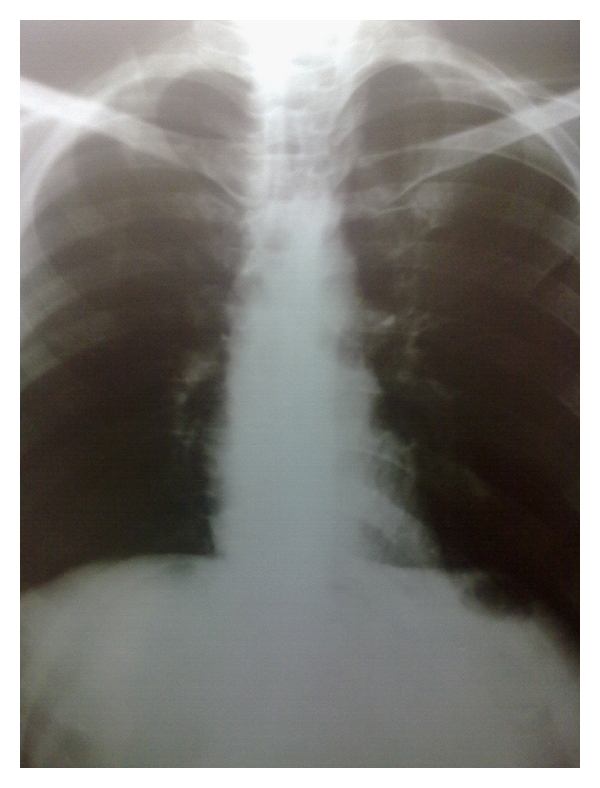
Chest X-ray.

**Figure 5:**
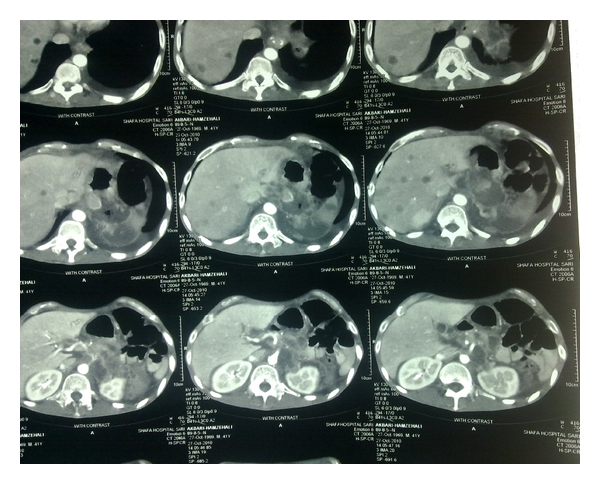
Abdominal CT Scan with contrast.

 A 41-year-old man came to orthopedic ward with the chief complaint of tender and painful lump on the anteromedial surface of his tibia in the proximal third which had been growing during previous 6 months. The patient was very cachectic and had the history of proximal gastrectomy and gastrojejunostomy which had been done 16 months ago due to gastric carcinoma accompanied with six sessions of chemotherapy and radiotherapy. In pathologic evaluation of the tumor in this operation, moderately differentiated adenocarcinoma with an-epithelial to-serosal extension and no lymph node involvement had been reported. In physical examination of the extremity, a firm and tender lump was seen with a diameter of 6 ∗ 4 cm which was firmly attached to the anteromedial surface of tibia. In simple X-ray an interesting feature was the slight thinning of the anterior cortex without cortical destruction which made the diagnosis of metastatic a bit unlikely (Figure 1(a) and 1(b)). In whole body bone scan with Tc-99 m MDP, the lesion showed activity in all 3 phases and was the only site of skeletal involvement (Figure 2). The patient went through excisional biopsy and the result of microscopic evaluation was metastatic adenocarcinoma (Figure 3(a) and 3(b)). Chest X-ray of patient was normal (Figure 4). But abdominal CT Scan with contrast showed some metastatic lesions in liver (Figure 5).

## 3. Discussion

Puri et al. in their report of three cases of gastric cancer presenting with distant metastasis, describe a case of metastasis to the left forearm in the form of soft tissue lump and another case of fibular metastatic lesion [3]. Kammori et al. in their case report presented a 49 years old man who suffered from metastatic involvement of seventh cervical vertebra 9 years after total gastrectomy for gastric cancer [4]. Ichiyoshi et al. evaluated the results of 503 cases of early gastric cancer and found 3.4% rate of recurrence consisting of liver, lung, and bone metastasis [5]. In the report of Sano et al. of the long-term followup of 1475 cases of early gastric cancer, twenty cases (1.4%) of recurrence were found including 14 cases of bony metastasis [6]. Based on literature, it seems that although bone metastasis is a rare clinical finding in gastric cancer. It may be due to underestimation and using whole-body bone scan can detect significant percentage of bone metastasis. Choi et al. investigated the bone scan of 234 cases of advanced gastric carcinoma out of 17176 total cases of gastric carcinoma. 106 cases of bone metastasis were found among these cases [7].

## 4. Conclusion

Even though metastasis to bone is much more common in the axial skeleton and the roots of extremities, we should still expect to see them in unusual sites and from those cancers not so notorious in invasion to bone from.
